# Seasonality in the relationship between equatorial-mean heat content and interannual eastern equatorial Atlantic sea surface temperature variability

**DOI:** 10.1007/s00382-021-06116-w

**Published:** 2022-01-14

**Authors:** Kyle J. Turner, Natalie J. Burls, Anna von Brandis, Joke Lübbecke, Martin Claus

**Affiliations:** 1 Department of Atmospheric, Oceanic, and Earth Sciences, Center for Ocean-Land-Atmosphere Studies, George Mason University, 4400 University Drive, Fairfax, VA 22030 USA; 2 GEOMAR Helmholtz Centre for Ocean Research Kiel, Düsternbrooker Weg 20, 24105 Kiel, Germany; 3 Christian-Albrechts-Universität zu Kiel (CAU), Christian-Albrechts-Platz 4, 24118 Kiel, Germany

**Keywords:** Tropical Atlantic, Interannual variability, Heat content, Atlantic Niño

## Abstract

Interannual sea surface temperature (SST) variations in the tropical Atlantic Ocean lead to anomalous atmospheric circulation and precipitation patterns with important ecological and socioeconomic consequences for the semiarid regions of sub-Saharan Africa and northeast Brazil. This interannual SST variability is characterized by three modes: an Atlantic meridional mode featuring an anomalous cross-equatorial SST gradient that peaks in boreal spring; an Atlantic zonal mode (Atlantic Niño mode) with SST anomalies in the eastern equatorial Atlantic cold tongue region that peaks in boreal summer; and a second zonal mode of variability with eastern equatorial SST anomalies peaking in boreal winter. Here we investigate the extent to which there is any seasonality in the relationship between equatorial warm water recharge and the development of eastern equatorial Atlantic SST anomalies. Seasonally stratified cross-correlation analysis between eastern equatorial Atlantic SST anomalies and equatorial heat content anomalies (evaluated using warm water volume and sea surface height) indicate that while equatorial heat content changes do occasionally play a role in the development of boreal summer Atlantic zonal mode events, they contribute more consistently to Atlantic Niño II, boreal winter events. Event and composite analysis of ocean adjustment with a shallow water model suggest that the warm water volume anomalies originate mainly from the off-equatorial northwestern Atlantic, in agreement with previous studies linking them to anomalous wind stress curl associated with the Atlantic meridional mode.

## Introduction

Sea surface temperature (SST) variations in the tropical Atlantic Ocean influence local atmospheric circulation patterns and precipitation in neighboring African and South American countries (Xie and Carton 2004). Such climate impacts carry important ecological and socioeconomic consequences in the semiarid regions of sub-Saharan Africa and northeast Brazil, which have a strong dependence on rain-fed agriculture (Carton and Huang 1994; Lutz et al. 2015). Given the influence of tropical Atlantic variability (TAV) on surrounding regions, a better understanding of TAV and its predictability is of significant interest.

Eastern equatorial Atlantic (EEA) SSTs are governed primarily by the seasonal cycle, but also exhibit variability on interannual to decadal time scales. Variability on these time scales is characterized by two primary modes, a meridional and zonal mode. The Atlantic meridional mode (hereafter abbreviated as AMM) features an anomalous cross-equatorial SST gradient that peaks in boreal spring (March–April–May; MAM) and is sustained by a positive atmosphere–ocean feedback between wind, evaporation and SST (WES feedback; Chang et al. 1997; Xie 1999; Amaya et al. 2017). Associated with the AMM is an anomalous displacement of the intertropical convergence zone (ITCZ) toward the warmer hemisphere (Chiang and Vimont 2004). The Atlantic zonal mode, also known as the Atlantic Niño due to its resemblance to the Pacific El Niño-Southern Oscillation (ENSO), appears with SST anomalies in the central to eastern equatorial Atlantic (6° S–2° N, 20° W–5° E) that peak in boreal summer (June–July–August; JJA) (Zebiak 1993; Chang et al. 2006). SST anomalies frequently exceed 1 °C during Atlantic Niño/Niña events (Xie and Carton 2004). Okumura and Xie (2006) identify a second zonal mode of variability with SST anomalies peaking in boreal winter (November–December; ND), which they term the Atlantic Niño II. According to Okumura and Xie (2006), the Atlantic Niño II exhibits 65% of the variance of the summer Atlantic Niño and is not correlated with the Atlantic Niño or ENSO. Relatively few studies have focused on the Atlantic Niño II within TAV.

The primary elements of the positive ocean–atmosphere Bjerknes feedback which is known to govern ENSO in the Pacific are active in the equatorial Atlantic as well (Okumura and Xie 2006; Keenlyside and Latif 2007; Deppenmeier et al. 2016; Lübbecke and McPhaden 2017). These elements include a coupling between EEA SST and zonal wind stress in the western basin, ocean sensitivity to surface stress depending on the vertical stratification, and the effect of subsurface on surface temperatures via upwelling and mixing. However, the strength of the feedback is significantly weaker in the Atlantic compared to the Pacific, and displays a stronger seasonal modulation (Burls et al. 2012). This may explain why Atlantic Niño events are phase-locked to boreal summer, and are weaker in amplitude, shorter in duration, and more frequent than Pacific El Niño/La Niña events (Keenlyside and Latif 2007; Carton and Huang 1994; Wang 2006; Burls et al. 2012).

Richter et al. (2012) distinguish between “canonical” and “non-canonical” Atlantic Niño events. Canonical events are consistent with ENSO-like dynamics, with rapidly-developing warm EEA SST anomalies in JJA preceded by westerly wind stress anomalies in MAM that inhibit upwelling in the Atlantic cold tongue region (3° S–3° N, 15°–5° W). Non-canonical events, on the other hand, show a slow evolution of EEA SST anomalies over the course of the year, preceded rather by easterly wind anomalies on the equator. Further, non-canonical events are associated with strong positive SST anomalies in the northern tropical Atlantic (NTA; 10°–20° N, 40°–10° W), and are driven by zonal and meridional advection, while canonical events are associated with weak negative anomalies in the NTA region, and are dominated by vertical advection.

The role of warm water volume (WWV) recharge/discharge of the equatorial Atlantic for TAV remains poorly understood, largely due to considerable uncertainties and inconsistencies in subsurface condition estimates across reanalysis products in the Atlantic (Zhu et al. 2012a, b). Using observed equatorial (3° S–3° N) Atlantic sea surface height (SSH) as a proxy for upper ocean heat content (HC), Ding et al. (2010) show a correlation between local equatorial HC and EEA SST, with a maximum correlation when local SSH anomalies lead SST by about 2 months. According to Ding et al. (2010), SSH, thermocline (20 °C isotherm) depth, and local HC are all closely related due to the two-layered nature of the tropical ocean. Bunge and Clarke (2009) and Hu et al. (2013) find that equatorial Atlantic upper ocean temperature variability is dominated by two leading modes, referred to as the “tilt” and “WWV” modes, representing changes in the east–west slope (zonally asymmetric) and mean depth (zonally symmetric) of the equatorial thermocline, respectively. These modes are analogous to similarly named modes defined originally for ENSO by Meinen and McPhaden (2000). While the tilt mode is driven mainly by zonal wind stress fluctuations in the southwestern tropical and equatorial Atlantic, changes in the WWV mode are driven by equatorial heat convergence that Hu et al. (2013) trace back to HC anomalies in the northwestern tropical Atlantic. Similar to the observationally based findings of Foltz and McPhaden (2010), anomalous wind forcing associated with the AMM was found by Hu et al. (2013) as being responsible for generating these off-equatorial HC anomalies. Hu et al. (2013) show that the WWV and tilt modes are to some extent connected, with WWV changes leading tilt changes. Their findings are consistent with other studies linking changes in tropical Atlantic oceanic and atmospheric conditions associated with the AMM to the development of the zonal mode in the equatorial Atlantic (Servain et al. 1999; Murtugudde et al. 2001; Foltz and McPhaden 2010; Zhu et al. 2012a, b; Burmeister et al. 2016; Martín-Rey and Lazar  2019).

While these previous studies have shown an influence of equatorial recharge on the development of EEA SST anomalies in boreal summer, the seasonality in the strength of this relationship has not been fully investigated. In this study we address this through (1) an analysis of seasonally stratified cross-correlations between EEA SST and equatorial WWV across both satellite SSH observations and a range of reanalysis products; and (2) an event-based analysis of ocean adjustment by means of a shallow water model. Our results suggest that while WWV changes do occasionally play a role, the tilt mode dominates the development of Atlantic Niño summer events. WWV changes do, however, contribute more consistently to Atlantic Niño II, i.e. boreal winter events.

The remainder of the paper is organized as follows. Section 2 gives a description of the different data sources and methods used in this investigation. Section 3 presents the primary results of the study, including an analysis of both the long-term temporal relationship between equatorial Atlantic SSH/WWV and EEA SST and the role of upper ocean HC recharge on the development of individual warm and cold events over the course of the year. Results are summarized and discussed in Sect. 4.

## Data and methods

### Observational data

For observed SST, two gridded data sets are utilized: The NOAA 1/4° daily Optimum Interpolation SST version 2 (OISSTv2) (Banzon et al. 2016) and the monthly Met Office Hadley Centre’s 1° Sea Ice and Sea Surface Temperature data set (HadISST1.1) (Rayner et al. 2003). The former is analyzed for the period 1982–2016, and the latter for the period 1980–2016. Monthly-averaged fields are computed from the daily OISST data over the time period mentioned. It is noted that a recent analysis by Chelton and Risien (2016) identifies a number of problems and discontinuities in the HadISST1.1 data set. However, Chelton and Risien (2016) conclude that these problems would not significantly impact studies of climate variability on interannual to decadal time scales and the results presented here appear to be robust across both the OISSTv2 and HadISST1.1 datasets. Global 1/4° gridded daily-mean SSH data are obtained from the Copernicus Marine and Environmental Monitoring Service (CMEMS; http://marine.copernicus.eu/) for the period 1993–2015. These multi-mission satellite altimeter data were previously distributed by Archiving, Validation, and Interpretation of Satellite Oceanographic data (AVISO) and are thus referred to as AVISO SSH throughout this manuscript. Monthly-averaged fields are computed from the daily SSH data and the monthly climatology is removed.

### Reanalysis products

In addition to observational data sets, output from four different ocean reanalysis products are used to calculate equatorial SSH, WWV, HC, and EEA SST anomalies. Monthly mean potential temperature and SSH relative to the geoid from the NCEP Global Ocean Data Assimilation System (GODAS) reanalysis, for the period 1980–2016, are obtained from the Physical Sciences Division of NOAA's Earth System Research Laboratory (ESRL) in Boulder, Colorado, USA (http://www.esrl.noaa.gov/psd/). GODAS data have a spatial resolution of 1/3° latitude by 1° longitude with 40 vertical levels. Temperature and SSH from the Simple Ocean Data Assimilation version 2.2.4 (SODA2.2.4) for the period 1980–2008 are obtained from IRI/LDEO Climate Data Library (http://iridl.ldeo.columbia.edu/) (Carton et al. 2005). SODA2.2.4 data are monthly with a horizontal resolution of 1/2° and 40 vertical levels. Monthly interpolated potential temperature and SSH from the third release of the Estimating the Circulation and Climate of the Ocean reanalysis version 4 (ECCOv4), covering 1992–2015, are obtained from the product website (www.ecco-group.org). These data are interpolated onto a regular grid with 1/2° resolution and 50 depth levels. Lastly, potential temperature and SSH from the European Centre for Medium-Range Weather Forecasts (ECMFW) Ocean ReAnalysis System 4 (ORAS4) for the period 1980–2015 are obtained from the University of Hamburg’s Integrated Climate Data Center (ICDC; http://icdc.cen.uni-hamburg.de/) (Balmaseda et al. 2013). ORAS4 data are monthly with a 1° spatial resolution (equatorially refined to 1/3°) and 42 vertical levels. Multi-product means (MPM) of equatorial SSH, WWV, and EEA SST anomalies are calculated from the four reanalysis products over the period 1980–2016.

### Shallow-water model

A linear shallow water model (SWM) is employed to examine the purely wind-driven component of anomalous EEA SST and WWV in the equatorial region (Greatbatch et al. 2012). The reduced gravity and the depth of the modeled interface at rest are set such that the gravity wave speed is 1.5 ms^−1^ which corresponds to that of the second baroclinic mode in the tropical Atlantic (Kopte et al. 2018). The model domain extends from 62.5° W to 15° E and from 20° S to 20° N with solid walls at the northern, southern, and western boundary. To prevent spurious propagation of Kelvin waves along these boundaries, sponge layers are applied to the momentum equations. Dissipation in the interior of the model domain is parameterized by a Laplacian diffusion of momentum using a lateral eddy viscosity of 300 m^2^ s^−1^ (Claus et al. 2014). The SWM is forced with monthly wind stress from ERA Interim global atmospheric reanalysis for the period 1979–2011 (Berrisford et al. 2011). The wind stress data have been linearly interpolated from a horizontal resolution of 3/4° to 1/2°, where only ocean grid points of the source grid where used to avoid influences from continental wind stress. Interface displacement anomalies are calculated relative to monthly means over the period 1984–2011. The period 1979–1984 is influenced by the spin-up of the SWM and is thus neglected. The interface displacement anomaly is a proxy for the thermocline displacement anomaly or warm water depth (WWD), and is referred to as such in the following.

### Index regions and associated calculations

**Fig. 1:**
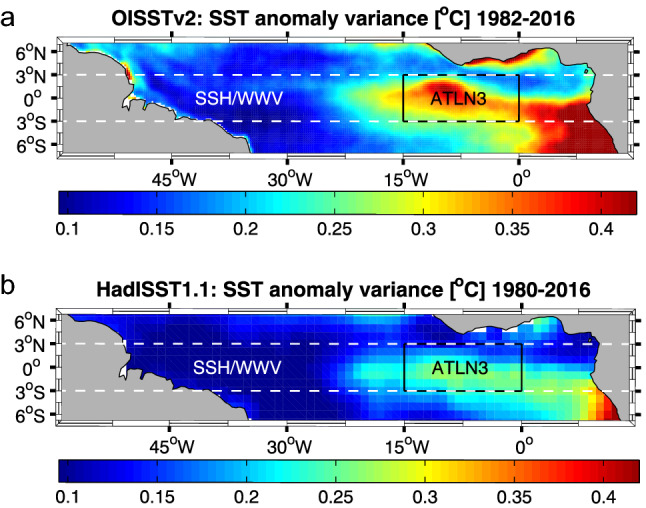
SST anomaly variance over 8°S-8°N, 60°W-15°E from **a** OISSTv2 (1982–2016) and **b** HadISST1.1 (1980–2016)

Equatorial Atlantic SSH, WWV, and HC are integrated over 3° S–3° N, 60° W–15° E (Fig. 1). WWV is calculated as the volume integral above the 20 °C isotherm, consistent with Bunge and Clarke (2009), Ding et al. (2010), and Hu et al. (2013). HC is defined as the volume average of temperature from the surface to 400 m depth. Anomalous EEA SST events are classified using the ATLN3 index region (3° S–3° N, 15° W–0°) as defined by Lutz et al. (2013). Note that the ATLN3 region differs slightly from the Atl3 index (3° S–3° N, 20° W–0°) used by Keenlyside and Latif (2007) and the Atlantic Niño II index (3° S–3° N, 15°–5° W) introduced by Okumura and Xie (2006), and later renamed the Atlantic cold tongue index (ACT1) by Richter et al. (2012). ATLN3 SST anomalies are calculated relative to monthly means over the full time periods of each observational and reanalysis data set described above. Atlantic Niño/Niña conditions are determined by finding where the detrended, area-average ATLN3 SST anomaly in JJA exceeds one standard deviation for at least 1 month. Likewise, Atlantic Niño II/Niña II conditions are determined by the same criteria but for ND. Only years where both observational SST data set (HadISST 1.1 or OISST v2) indicate Atlantic Niño/Niña II event conditions are included in our composite analysis.

### Lag-lead correlations

The temporal relationship between equatorial SSH/WWV and ATLN3 SST anomalies/AMM events is examined by calculating monthly stratified cross-correlations. The timeseries are detrended and a 3-month running mean is applied to the data before the correlation analysis, and only values exceeding the 95%-significance level according to a two-tailed student t-test are shown. We also have tested the implications of applying a < 7-year highpass filter. Applying this filtering leads to similar, yet stronger, correlation patterns.

## Results

### Seasonality of relationship between HC and SST anomalies

**Fig. 2:**
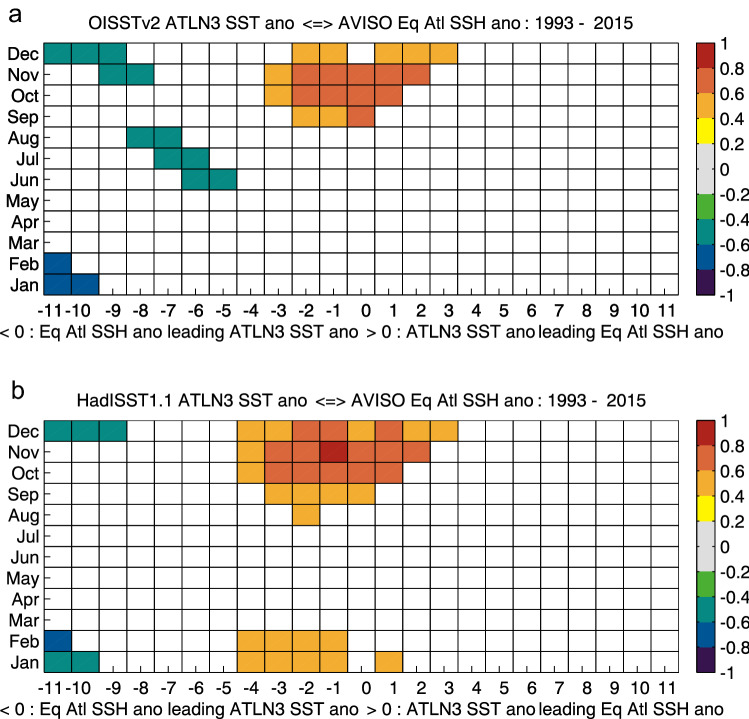
Lag-lead correlations of observed detrended ATLN3 SST anomalies with observed detrended equatorial Atlantic SSH anomalies: **a** OISSTv2 vs. AVISO (1993–2015), **b** HadISST1.1 vs. AVISO (1993–2015)

Figure 2a, b show the seasonal dependence in the correlation between anomalous equatorial Atlantic SSH as a proxy for basin mean equatorial heat content and observed ATLN3 SST anomalies. In both OISSTv2 and HadISST1.1, we see that it is mainly anomalous EEA SST between September and December that is significantly correlated with equatorial SSH anomalies, with correlations of 0.6–0.8 from September–November in OISSTv2 and October–December in HadISST1.1, for SSH leading by 3 months to lagging by 2 months. The highest correlation of 0.81 is present in HadISST1.1 in November, with SSH leading SST by 1 month. Negative correlations of − 0.45 to − 0.7 from December–February are present in both data sets at a 9–11-month lead. OISSTv2 shows additional negative correlations of − 0.44 to − 0.51 in JJA at a 5–8-month lead time which are absent in HadISST1.1. HadISST1.1 also exhibits positive correlations of 0.44–0.57 in January and February at a 4-month lead to a 1-month lag time. In both observational sources, significant positive correlations are predominantly absent during JJA, the peak phase of the Atlantic Niño. To further investigate this pronounced seasonality, we will concentrate on summer and winter events separately and also take into account information from several reanalysis products.

**Fig. 3:**
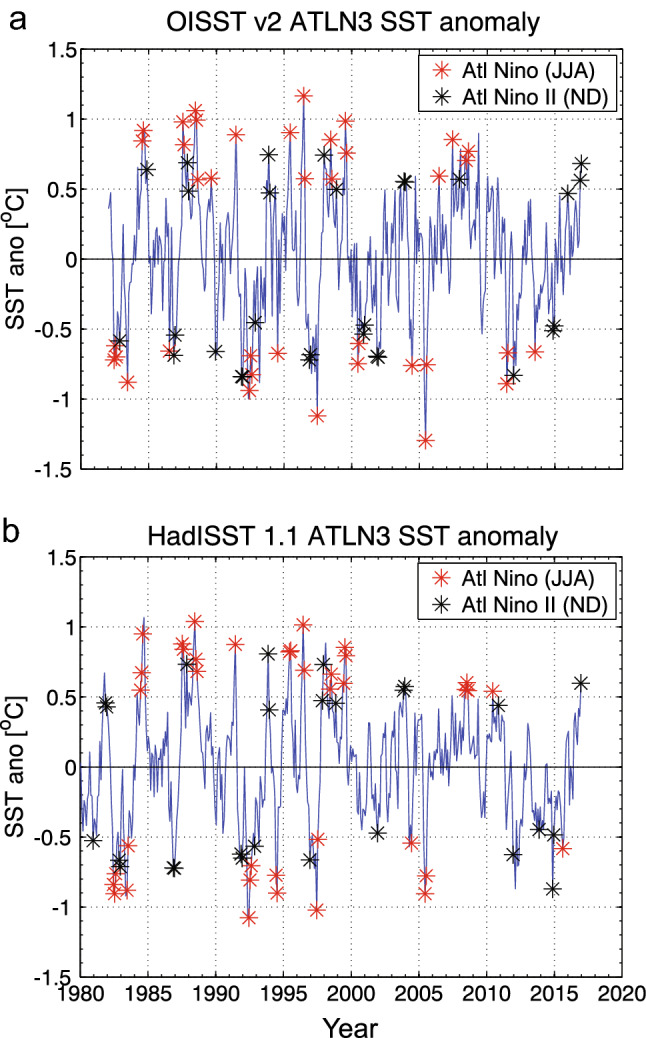
Monthly detrended ATLN3 SST anomalies from **a** OISSTv2 and **b** HadISST1.1 with the Atlantic Niño/Niña (JJA) events in Table 1 superimposed as red stars and the Atlantic Niño II/Niña II (ND) events in Table 2 superimposed as black stars. Markers show the individual months of JJA or ND that exceed one standard deviation of JJA or ND respectively

Time series of ATLN3 SST anomalies from OISSTv2 and HadISST1.1 are shown in Fig. 3. Individual months in JJA and ND that meet the Niño/Niño II event criteria are indicated as red and black stars respectively. As expected, the largest SST anomalies occur in JJA, with ND anomalies exhibiting comparatively lower variance.

**Fig. 4:**
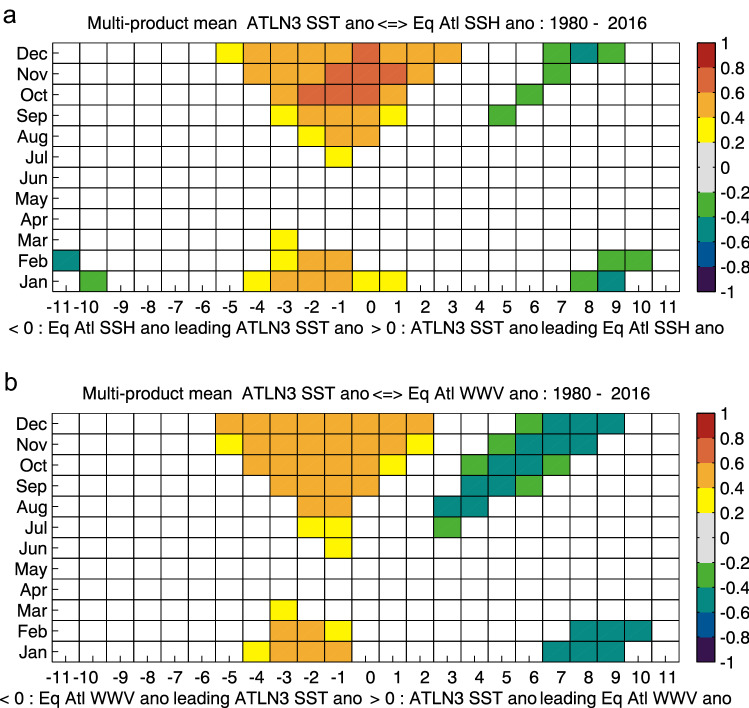
Lag-lead correlation of reanalysis multi product mean (MPM) detrened ATLN3 SST anomalies with **a** MPM detrended equatorial SSH anomalies, and **b** MPM detrended equatorial WWV anomalies for the period 1980–2016

Lag-lead correlations between the multi product mean (MPM) ATLN3 SST anomalies and MPM SSH anomalies show the strongest positive correlations of 0.61–0.69 in October–December, with a 2-month lead to a 1-month lag time (Fig. 4a), similar to the correlations of observed ATLN3 and SSH anomalies (Fig. 2). A similar correlation pattern is seen when using WWV as an alternative metric for equatorial recharge, although with weaker correlations in October–December compared with SSH (Fig. 4b).

**Fig. 5:**
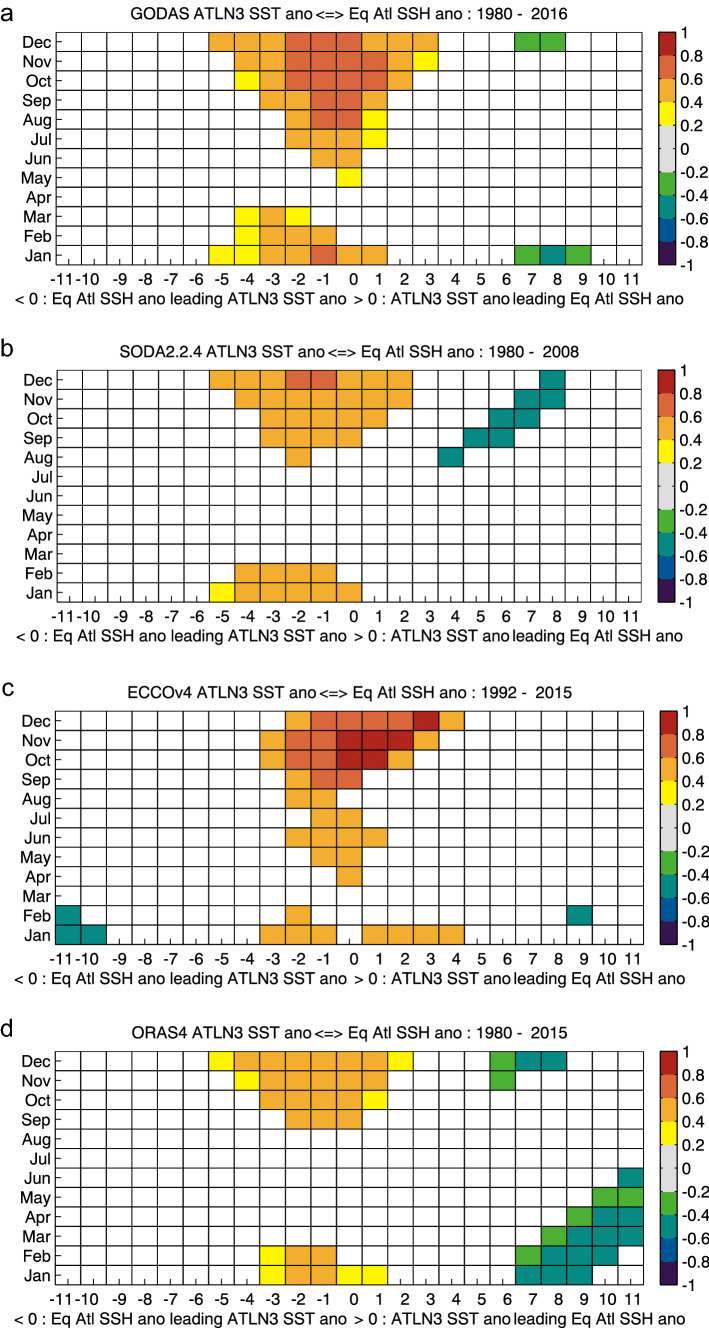
Lag-lead correlations of detrended ATLN3 SST anomalies and detrended equatorial SSH anomalies from individual reanalyses: **a** GODAS (1980–2016), **b** SODA2.2.4 (1980–2008), **c** ECCOv4 (1992–2015), **d** ORAS4 (1980–2015)

While they all capture the broad September–December peak in ATLN3 SST and SSH correlations, considerable variability is seen in the strength of this relationship between the four individual reanalysis products analyzed (Fig. 5), and similarly WWV (not shown). These differences across reanalysis products might be due to the impact that model errors can have on the link between subsurface and surface variability (e.g. Ding et al. 2015). ECCOv4 shows the highest positive correlations in October–December, at a 2-month lead to 3-month lag, and also shows significant correlations during other months of the year. GODAS likewise exhibits the strongest correlations in August–December, and significant correlations in boreal spring and summer months. SODA2.2.4 and ORAS4 show little-to-no correlation in spring and summer months (March–August), with positive correlations from 0.40 to 0.62 from September to February at a 1-month lag up to a 4-month lead time. Despite their differences, they all agree on the seasonality of the relationship with higher correlations in the boreal winter than summer months.

The results from both the observational data and the reanalysis products therefor suggest that the WWV mode plays a more consistent role in the development of Atlantic Niño II events in boreal winter compared to summer Atlantic Niño events. To further investigate this relationship, we will focus on the development of individual summer and winter Atlantic Niño and Niña events.

### Individual Atlantic Niño II events

From 1980 to 2016, both OISSTv2 and HadISST1.1 data sets indicate nine Atlantic Niño events (1984, 1987, 1988, 1991, 1995, 1996, 1998, 1999, and 2008) and seven Atlantic Niña events (1982, 1983, 1992, 1994, 1997, 2004, and 2005). Both observational sources agree on six Atlantic Niño II events (1987, 1993, 1997, 1998, 2003, and 2016) and eight Atlantic Niña II events (1982, 1986, 1991, 1992, 1996, 2001, 2011, and 2014) over the same period. Reanalyses are mostly in agreement with observations over these years, with the exception of the 1998 Niño II and 2014 Niña II events, which are not captured by all of the reanalysis products. An overview of all events and their representation in the different data sets is given in Tables 1 and 2.

**Table 1:** Atlantic Niño/Niña (JJA) events (1980–2016)

Year	HadISST 1.1	OISST v2	GODAS	SODA 2.2.4	ECCO v4	ORA S4
1982 (cold)	X	X		X	–	X
1983 (cold)	X	X		X	–	X
1984 (warm)	X	X	X		–	
1986 (cold)		X		X	–	X
1987 (warm)	X	X	X		–	X
1988 (warm)	X	X	X	X	–	X
1989 (warm)		X			–	
1991 (warm)	X	X	X	X	–	X
1992 (cold)	X	X	X	X	X	X
1994 (cold)	X	X	X	X		
1995 (warm)	X	X	X	X	X	X
1996 (warm)	X	X	X	X	X	X
1997 (cold)	X	X	X	X	X	X
1998 (warm)	X	X	X	X	X	X
1999 (warm)	X	X	X	X	X	X
2000 (cold)		X	X		X	
2004 (cold)	X	X	X		X	X
2005 (cold)	X	X	X	X	X	X
2006 (warm)		X	X		X	X
2007 (warm)		X		X	X	X
2008 (warm)	X	X	X	X	X	X
2010 (warm)	X		X	–		
2011 (cold)		X		–		X
2013 (cold)		X		–		
2015 (cold)	X			–		

A X indicates the presence of an event in the respective dataset

Empty cells indicate that the event criteria is not met in the respective dataset and dashes indicate data not available

**Table 2:** Atlantic Niño II/Niña II (ND) events (1980–2016)

Year	HadISST 1.1	OISST v2	GODAS	SODA 2.2.4	ECCO v4	ORA S4
1980 (cold)	X	–			–	
1981 (warm)	X	–			–	X
1982 (cold)	X	X	X	X	–	X
1984 (warm)		X			–	
1986 (cold)	X	X	X	X	–	X
1987 (warm)	X	X			–	X
1989 (cold)		X			–	
1991 (cold)	X	X	X	X	–	X
1992 (cold)	X	X	X		–	X
1993 (warm)	X	X	X	X	X	X
1996 (cold)	X	X	X	X	X	X
1997 (warm)	X	X	X	X	X	X
1998 (warm)	X	X				
2000 (cold)		X		X	X	
2001 (cold)	X	X	X	X	X	X
2003 (warm)	X	X	X	X	X	X
2004 (cold)				X		X
2007 (warm)		X				
2010 (warm)	X		X	–	X	
2011 (cold)	X	X	X	–	X	X
2013 (cold)	X			–		
2014 (cold)	X	X		–		
2015 (warm)		X	X	–		
2016 (warm)	X	X	X	–	–	–

An X indicates the presence of an event in the respective dataset

Empty cells indicate that the event criteria is not met in the respective dataset and dashes indicate data not available

The seasonally stratified cross-correlations indicated a stronger connection between WWV and SST anomalies in boreal fall and winter. Therefore, here we will exemplarily show the evolution of one Atlantic Niño II warm and cold event each to elucidate the role of WWV in these events.

**Fig. 6:**
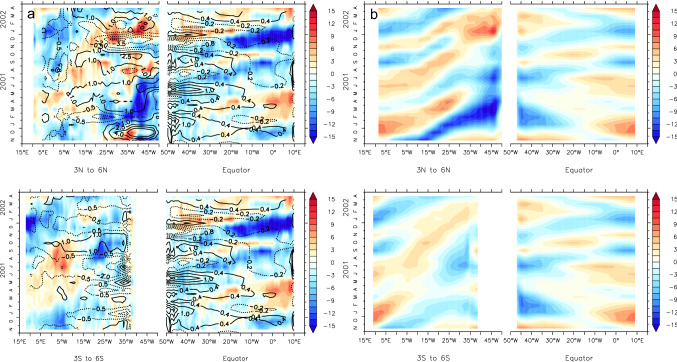
Longitude-time diagrams of the WWD anomalies from ORAS4 (**a**) and thermocline displacement from the SWM (**b**) for the 2001 Atlantic Niña II event. The longitude axes of the off-equatorial regions are reversed. In **a** anomalies of wind stress curl (for 3° N/S to 6° N/S) and zonal wind stress (for 2° N to 2° S) from ERA-Interim are overlaid

As an example for a cold event, we look at the 2001 Atlantic Niña II, which shows up in both observational data sets and all four reanalysis products (Table 2). Longitude-time diagrams of the WWD anomalies during this cold event suggest that it is connected to WWV anomalies in the northwestern tropical Atlantic via Rossby and Kelvin wave propagation (Fig. 6). The evolution is shown here for ORA-S4 but looks similar in the other reanalysis products. A westward propagating negative WWV anomaly originating around 25° W to the north of the Equator reaches the western boundary in boreal summer (Fig. 6a). A subsequent upwelling Kelvin wave along the Equator leads to a shoaling of the thermocline in the EEA, which is reinforced by a second eastward propagating upwelling signal about a month later (Fig. 6a). There is an indication that the second upwelling Kelvin wave might be connected to negative WWV anomalies to the south of the equator (Fig. 6a). The fact that this evolution is well reproduced by the SWM (Fig. 6b) points to the dominant role of wind-driven dynamics. Indeed, the shoaling of the thermocline and corresponding negative WWV anomaly in the northwestern tropical Atlantic in boreal winter and spring follows a pronounced positive wind stress curl anomaly. Positive zonal wind stress anomalies, i.e. stronger easterly trade winds, in the western equatorial Atlantic in early summer and late fall likely further enhanced the upwelling equatorial Kelvin wave signal (Fig. 6a).

**Fig. 7:**
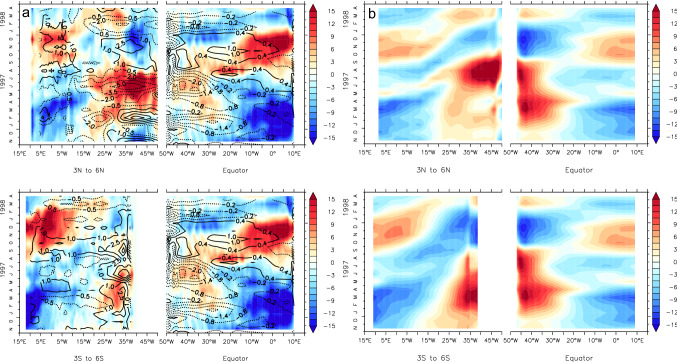
Same as Fig. 6 but for 1997

As an example for a warm event, we consider the 1997 Atlantic Niño II. This event is also common to all data sets used in this study (Table 2). This event is characterized by a strong build-up of heat in the western basin, both north and south of the equator, in spring and summer (Fig. 7). In particular the anomaly to the north of the Equator is clearly related to a strong negative wind stress curl anomaly. The deepening of the thermocline then propagates towards the eastern equatorial basin in late boreal summer 1997, intensifying there to reach its maximum in fall and winter. Also in this case, western equatorial wind stress anomalies, namely a weakening of the easterly trades in boreal summer 1997, likely enhanced the signal. Again, the SWM is for the most part able to reproduce this evolution. In many ways the WWD evolution in 1997 resembles that of its seasonal evolution (Ding et al. 2009) but opposite in sign, giving some support to the perspective that Atlantic interannual variability is largely a modulation of the seasonal cycle (Burls et al. 2011, 2012).

In both cases, anomalous WWV in the (north)western tropical Atlantic acts as a precursor of the Atlantic Niño/Niña II event. This confirms the importance of WWV changes in the development of boreal winter SST anomalies in the EEA. The same holds true for other events, e.g. the 1991 cold event, but it is worth noting that this is not common to all Atlantic Niño/Niña II events indicating that feedbacks associated with local surface and wind stress forcing can also contribute to event development. Similarly, it has been found that feedbacks other than the thermocline and advective feedbacks play a larger role in the development boreal summer Atlantic Niño/Niña feedbacks relative to its Pacific counterpart, ENSO (Chang et al. 2006; Nnamchi et al. 2015).

### Composing Atlantic Niño II events

**Fig. 8:**
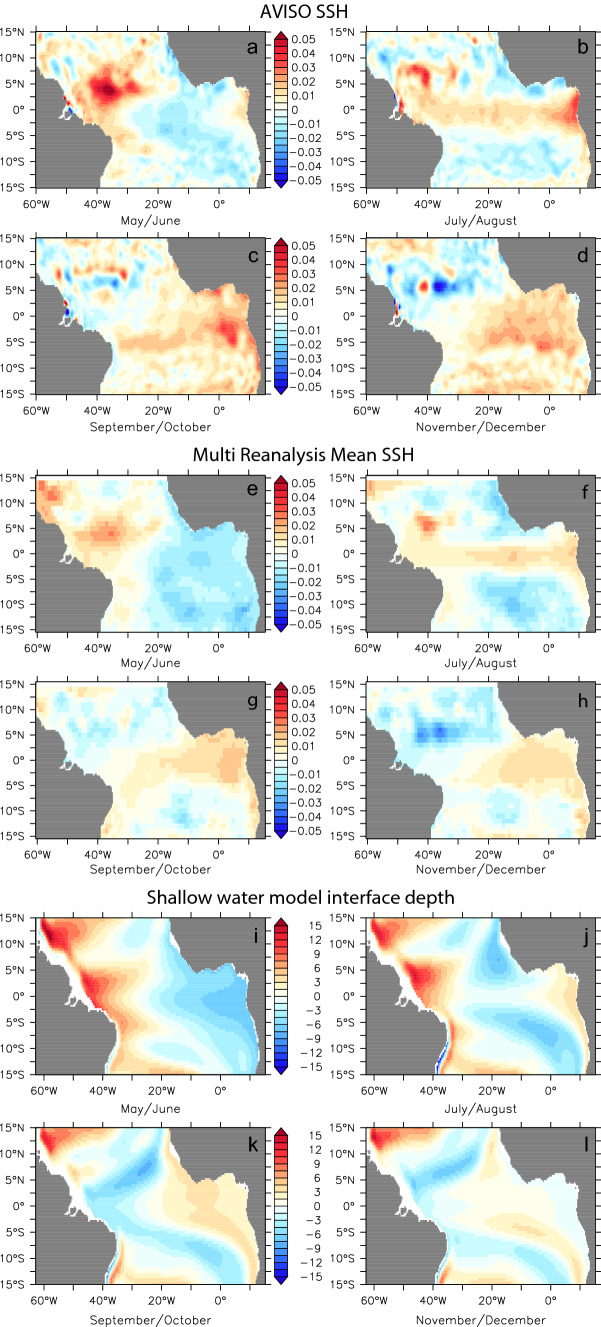
Composite anomalies of SSH (in m) from AVISO (**a**–**d**) and Multi Reanalysis mean (**e**–**h**) and thermocline displacement (in m) from the shallow water model (**i**–**l**) for warm Atlantic Niño II years (1997, 1998, 2003 for **a**–**d**; 1987, 1993, 1997, 1998, 2003 for **e**–**l**)

**Fig. 9:**
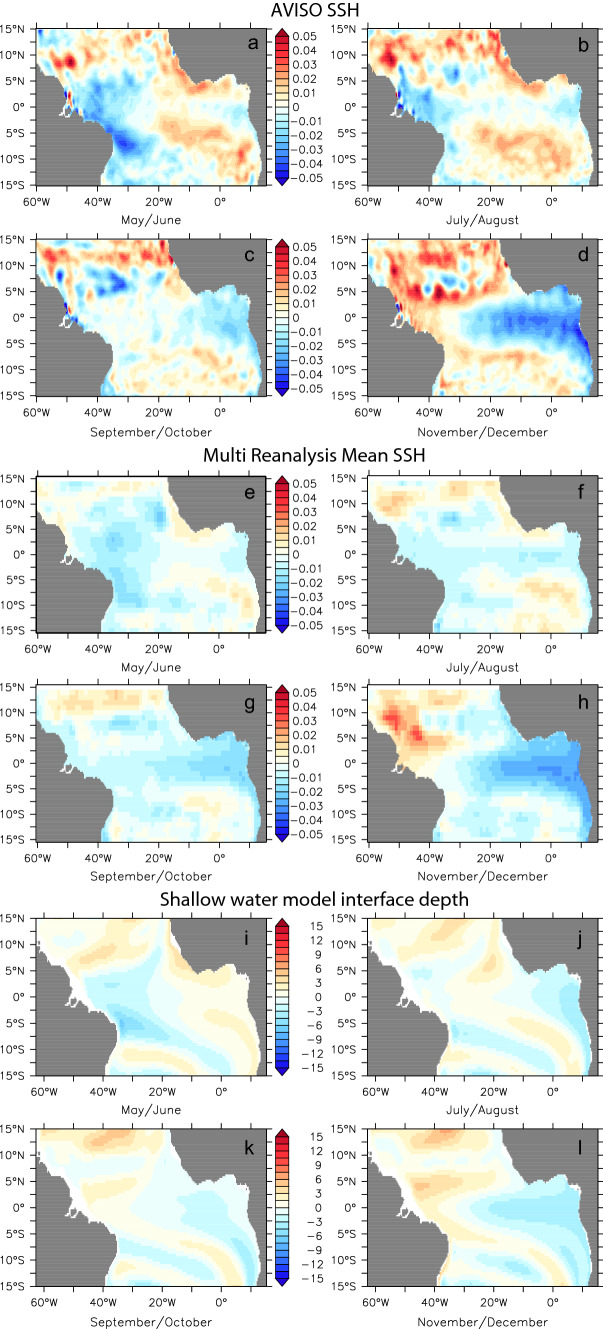
Composite anomalies of SSH (in m) from AVISO (**a**–**d**) and Multi Reanalysis mean (**e**–**h**) and thermocline displacement (in m) from the shallow water model (**i**–**l**) for cold Atlantic Niña II years (1996, 2001, 2011 for **a** –**d**; 1986, 1991, 1992, 1996, 2001 and 2011 for **e**–**l**)

Composites of SSH (and SWM interface depth) for warm Atlantic Niño II years (Fig. 8) and cold Atlantic Niña II years (Fig. 9) show a common May–December evolution that is generally consistent across the AVISO, multi reanalysis mean and SWM datasets. Warm event composites show the build-up of heat content in boreal spring in the northwestern tropical Atlantic (Fig. 8a, e, i). This signal begins propagating to the EEA, with weak EEA anomalies emerging in summer (Fig. 8b, f, j), and further strengthening into boreal winter (Fig. 8c, d, g, h, k, l). The cold event composites (Fig. 9) depict roughly the opposite evolution, the main distinction being that the decrease of heat content in boreal spring occurs predominantly in the southwestern tropical Atlantic (Fig. 9a, e, i). Also the anomalies associated with the cold events appear to be stronger. That said, this distinction may be purely an artifact of the limited sample size of these composites. All in all, the composite analysis in Figs. 8 and 9 highlights a basin mode of wind-driven Rossby and Kelvin Wave adjustment that leads to the buildup/depletion of equatorial-mean heat content and subsequent Atlantic Niño II/Niña II events. The importance of ocean waves and in particular the reflection of Rossby waves into equatorial Kelvin waves in determining the structure and timing of the modes of Atlantic Equatorial variability has also recently been highlighted in a model study by Martín-Rey et al. (2019).

## Summary and discussion

While TAV on interannual timescales displays many characteristics that resemble Pacific ENSO dynamics, the mechanisms giving rise to interannual SST variability are more diverse with both dynamic and thermodynamic local air-sea interactions causing anomalous conditions (Chang et al. 2006; Lübbecke et al. 2018; Nnamchi et al. 2015, 2021). As a result, the role of equatorial recharge in the generation of Atlantic zonal mode warm events in boreal summer (JJA), referred to as Atlantic Niños, has been harder to ascertain than within its Pacific counterpart.

Previous studies have shown that the Bjerknes Feedback and tilt mode play a clear role in the generation of many boreal summer Atlantic Niño events (Keenlyside and Latif 2007; Deppenmeier et al. 2016; Lübbecke and McPhaden 2017). The smaller basin size allows the Atlantic to adjust more quickly to wind perturbations and as a result, oscillations in the tilt mode occur predominantly on seasonal timescales in the Atlantic (Burls et al. 2012). On the other hand, as mentioned in the introduction, some Atlantic Niño events are “non-canonical” and driven by zonal and meridional advection, rather than the Bjerknes Feedback under which vertical advection anomalies dominate the surface mixed-layer heat budget (Richter et al. 2012).

The other robust feature of ENSO events is equatorial recharge/the WWV mode. Ding et al. (2010) find that the relationship between equatorial recharge and interannual variability in eastern equatorial SST is considerably weaker in the Atlantic than in the Pacific, but they do not evaluate the seasonality in this relationship. The analysis we have undertaken addresses this gap and evaluates the seasonality in the relationship between equatorial HC and EEA SST anomalies. Our correlation analysis suggests that the dominant influence of equatorial HC anomalies is on the development of EEA SST between September to December—the Atlantic Niño II mode of Okumura and Xie (2006). For both an Atlantic Niño II cold and warm event, we show how, consistent with equatorial wave dynamics in a SWM, off-equatorial HC anomalies predominantly from the northwestern tropical Atlantic propagate to the western boundary and then eastward along the equator, giving rise to an anomalous SSTs in November–December. The origin of the HC anomalies in the off-equatorial northwestern Atlantic is in agreement with the results of Hu et al. (2013) who attributed this to anomalous wind stress curl associated with the Atlantic meridional mode.

It is interesting to note that while the overall relationship between JJA Atlantic Niño events and JJA equatorial recharge is weak across our 1980–2016 analysis period (Fig. 4), the relationship appears to be more consistent when considering only non-canonical Atlantic Niño events—a result consistent with zonal and meridional advection mechanism put forward by Richter et al. (2012). The non-canonical events during this period occurred in 1987, 1998, 2006 and 2010. Both the 1987 and 2010 events are associated with the largest JJA equatorial recharge values with the 1980–2016 time series and both 1998 and 2006 coincided with higher levels of recharge than any of the canonical event years (with the exception of 1998). Non-canonical events on the other hand display no consistent relationship with recharge giving rise to low correlations seen in Fig. 4 between ATLN3 SST anomalies and equatorial recharge for JJA.

In light of the robust relationship between October–November–December EEA SST anomalies and equatorial heat content at a several months lead, assessing the implications for the predictability of November–December–January SST in seasonal reforecasts merits attention in future work. In a recent study on seasonal predictions of tropical Atlantic climate variability, Counillon et al. (2021) indeed relate the skill they find in predicting the November to December SST variability to off-equatorial heat content anomalies.

## Declarations

### Availability of data and material

The analysis presented in this paper is based on previously published datasets the availability of which is described in the Data and Methods Section.
